# Consumer health information needs in China – a case study of depression based on a Social Q&A community

**DOI:** 10.1186/s12911-020-1124-1

**Published:** 2020-07-09

**Authors:** Wang Zhao, Peixin Lu, Siwei Yu, Long Lu

**Affiliations:** 1 School of Information Management, Wuhan University, Wuhan, China; 2 Guizhou Provincial People’s Hospital, Guiyang, China

**Keywords:** Health information, Social Q&a community, LDA

## Abstract

**Background:**

The social Q&A community quickly becomes a popular platform for consumers to find health information because of its convenience and interactivity.

**Methods:**

Based on the 10,861 depression questions collected in the Zhihu, the largest Q&A platform in China, we divided the healthy information needs description into nine categories with Latent Dirichlet Allocation (LDA). We also divided the healthy information needs type into Physiological, affective and cognitive needs based on the Wilson model.

**Results:**

The results show that the largest categories are depression symptom and social activities while the less concerned health information is prevention and medical insurance. More attention is paid to cognitive needs. We also find there is no strong correlation between attention and needs type.

**Conclusions:**

The purpose of this paper is to refine the consumer health information needs types to better understand the consumer health information characteristic in China.

## Background

### First source of health information

With the rapid development of China’s economy and the implement of “Healthy China” strategy [1], people have paid more and more attention to their own health. In health care, the rapid increase in health information on the Internet has led more patients to turn to the Internet as their first source of health information and to gain knowledge about their health before seeking a professional diagnosis [2–4]. In the 2013 Pew Internet survey, searching for health information was the third most popular online activity; 72% of respondents reported they had used the internet to search for health information in the past year [5]. In China, health and medical care ranks as the most popular scientific topic among Chinese netizens, with a query rate of 63.16% [6].

### Value of user information needs

Different from Baidu, Google and other traditional search engines, the question-and-answer web service provides users with a more convenient and specific platform for expressing complex needs. Users can not only look for basic information, but also seek social and emotional support from their unique social attributes [7]. With its interactive nature, the online community has quickly made the social question and answer service a platform for users to search for relevant information [8]. Therefore, research on network health community information, discover user information needs, and study the characteristics of user information needs, can better promote the operation and information services of the network healthy community.

### Depression and social Q&A platform

Depression is a state of low mood and aversion to activities. It can affect a person’s thoughts, behavior, motivation, feelings, and sense of well-being. Its main features include sadness, difficulty in thinking and concentration and a significant increase/decrease in appetite and time spent sleeping. People experiencing depression may have feelings of dejection, hopelessness and, sometimes, suicidal thoughts. It can either be short term or long term [9]. Depression is a common mental disorder. Globally, more than 300 million people of all ages suffer from depression [10]. It is different from other types of non-mental diseases. Which can be divided into subgroups based on different levels of social barriers. Patients are more inclined to seek health information through the online platforms. The social Q&A platform is one of the main channels for people with depression to exchange information and seek help because of their social attributes.

### Purpose of this study

In order to obtain the healthy information different type needs considering the healthy information description types, healthy information needs type and the correlation with the attention, this article focuses on the social Q&A community commonly used by health consumers to obtain healthy relevant information. Taking the largest social Q&A community in China as an example, we selected 10,862 questions and related records of depression on the platform of Zhihu. Using latent Dirichlet allocation (LDA) to obtain the type of health information needs, refines its demand types, realizes the measurement of user information need characteristics which can helps them better organize information resources, and enhances the efficiency of users’ access to health information.

## Literature review

### Health information needs in social media

According to the definition of the National Network of Libraries of Medicine, consumer health information is health and medical information related to the general public, patients and their families [11]. Network health information plays a huge role in consumer health services. Health information research based on the social media focuses on four aspects: health information behavior research, health information needs research, health information assessment and health information user research [12]. In terms of network health needs, the main focus is on the research on the content and characteristics of health information needs of different types of users. Valero-Aguilera B et al. found that women with breast cancer are interested in information on cure rates, survival rates, and the effects of disease on shape. While the urological cancer patients pay more attention on the information linked to their sex life, keeping healthy, and exercise [13]. Tsuya et al. analyzed the profiles and posts of users who were concerned about nine cancer-related accounts on Twitter to understand the similarities and differences between social media usage and health information needs of different cancer patients [14]. Hyun Jung Oh et al. surveyed and analyzed 291 respondents who used Facebook, and found that emotional support for people looking for health information on Facebook is more frequent than other support [15]. Ramo et al. used a combination of questionnaires and interviews to explore how young people are interested in quitting smoking through Facebook and how to motivate users to participate more [16].

### Health information needs in Q&a site

Research on the information needs of the network health community helps to understand consumer needs and improve the service level of the network healthy community. At present, the research on health information needs in the question and answer community is mainly based on Yahoo! Answers and the method mainly about content analysis. There are few studies on the health needs of the social Q&A community in China. Sun et al. examined different content, styles and emotion expression by using manual statistical methods and got the needs topics by taking eating disorder as an example [17]. Sanghee Oh et al. used a content analysis method to analyze the cancers and sexually transmitted diseases in Yahoo! Answers, and proposed a coding model for disease-related problems in social Q&A websites [18]. They also compared the college students in the USA and Korea using convenience sample, in order to understand the proportion and characteristics of college students looking for health information through social media [19]. Kim et al. also analyzed 1000 posts through content analysis methods to clarify the topic of question and the question time of married Korean women living in the USA who sought help for health and medical issues [20]. Yong Jeong Yi collected the Q&A of sexual health information on a social Q &A site to identify their needs and the factors with the best answers [21]. There is less research on other platforms and methods. Donghua Chen et al. proposed a knowledge representation framework based on authoritative knowledge sources in online health communities to meet information needs of online patients [22].

In summary, the current research on the information needs of the social question and answer community mainly focuses on the theme and the influence of the needs. Few studies focus on the systematic classification of the types of needs. At the same time, the research methods are mostly based on statistical analysis methods (such as manual annotation and content analysis). LDA (latent Dirichlet allocation) is widely used in text mining, including text topic recognition, categorization and similarity calculation in recent years [23]. There are very few studies on the application of LDA to find the health information topic on the social Q&A community. Therefore, this paper uses the combination of LDA and manual labeling to systematically encode and classify consumers’ health information needs with depression as an example.

## Methods

### Data source: Zhihu

According to the 41st “Statistical Report on the Development of China’s Internet Network” released by the China Internet Network Information Center (CNNIC), until December 2017, the number of users in Zhihu had increase most in social applications, from 7.6 to 14.6%. Ranked fourth in social application [24]. As the largest social Q&A platform in China, the latest operational data has been released by Zhihu recently: by the end of August 2018, the number of registered users has exceeded 200 million. In 2018, there were more than 80 million new users [25]. The number of followers on the topic of “health” and relative questions has exceeded 8.12 million and 690,000 respectively. In Zhihu, users are able to ask questions, discuss and interact with others on the site. There’re a variety of operations in Zhihu for an answer. In addition to responding, you can also agree, share, thank, collect, and consider it as no help. A question in Zhihu can be “followed” by users so that they would receive notifications about this question, e.g. when the question is answered.

### Data collection

In order to improve the efficiency of data collection and save computing resources, we scratched the questions(*N* = 10,861) under the topic of “depression” which were published from January 2018 to July 2018 using the Python. For each question, we collected the related information of the question, including headline, description, topic, time that the question was raised and the number of answers. What’s more, we collected the information about the questioner such as the number of followers, the number of answers for the question. In order to ensure the reliability and conciseness of information, we first removed the anonymous answers and questions. Then, we used manual screening to remove unrelated and duplicated questions. Duplicated questions are the questions that are described in different ways but talking about the same topics. For a question, two people decide whether to delete it. If the results of the two people were different, the third person made the final decision. For duplicated questions, we only selected the most informative one of them to represent the topic. All coders are the graduate student of medical. The final corpus for analysis includes 2130 questions.

### Coding scheme development

The data coding rules of the traditional network health community are mostly determined by manual methods. The researchers combine the existing thesaurus to adjust the coding rules according to the actual situation of the collected data, and finally form the topic division strategy, and then perform manual coding. This method is easy, but there are problems such as subjective standard differences between different coding personnel, time consuming operations and unobjective classification. This paper combined LDA and manual methods to determine the subject coding rules.

**Fig. 1:**
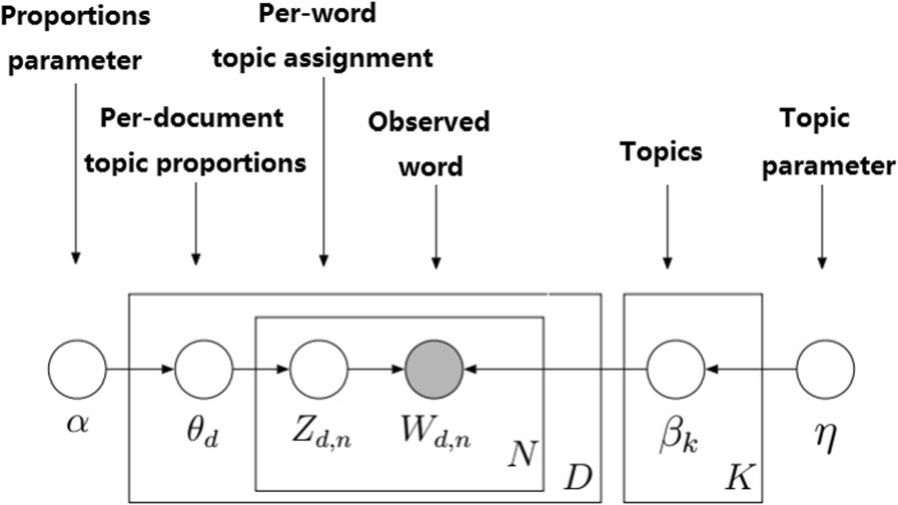
LDA model workflow

LDA (latent Dirichlet allocation) is an example of a topic model in natural language processing that was rediscovered independently by David Blei, Andrew Ng in 2003 [26]. LDA is a three-layer Bayesian probability model that contains documents, words and topics. The specific model is shown as Fig. 1 and Table 1.

**Table 1:** Parameter interpretation in LDA model

α	Dirichlet proportions parameter of ϴ
β	Dirichlet proportions parameter of φ
W_d,n_	The nth word in document d
Z_d,n_	The nth topic in document d
N	Number of words included in the document d
K	The number of topics
φ_k_	Represents the kth topic - word distribution
ϴ_m_	Represents the mth document - topic distribution

Each Q&A record is used as a document, and the LDA model is used to train. Finally, the keywords corresponding to the topics and themes are obtained. The training process is as follows:

#### Step 1: text segmentation

We used a jiebaR, an R package for Chinese text segmentation, keyword extraction and speech tagging, to do the initiatory text segmentation [27]. The default word segmentation system dictionary not enough, which led to unsatisfactory results. So, the author added the bio-dictionary and Sogou dictionary in the Sogou cell vocabulary to the user segmentation dictionary (user. Dict. utf8) in jiebaR, so that some of the word segmentation text Fixed words could be recognized. The Chinese stop word list (stop_word. utf8) was also added, which could help filter virtual words, auxiliary words, nonsense common words and punctuation marks.

#### Step 2: create document-word matrix

We used the tm package to process the word segmentation text [28], create a corpus for the list after the jieba word segmentation and create a document-word matrix (DTM). The constraint in the process of creating the matrix is the minimum frequency at which the word segment appears (minDocFreq) is 2, the frequency of occurrence is too low, there is no need to enter, the length of the word segmentation (word Lengths) is more than 2 characters (because the general single words in modern Chinese are not very meaningful, so the length is 1 character deleted. The elements in each matrix were weighted by word frequency (Tf) as a participle.

#### Step 3:select the topic number

LDA is unsupervised learning, so we used maximum likelihood estimation to optimize the number of topics selected.

#### Step 4:building an LDA model

LDA topic modeling for DTMs that had been segmented using topicmodels package.

Because this research involves the knowledge of the medical field in the social Q&A platform, only relying on the LDA classification cannot guarantee accuracy, so it is necessary to combine other information, such as PubMed [29], Anxiety and Depression Association of America [30] and other related literature, and eventually forming a depression theme information classification strategy.

According to the depression theme information classification strategy, descriptions of health information needs were divided into nine categories. According to Wilson’s information need model [31], we also divided the needs types into three categories: physical needs, emotional needs and cognitive needs. In order to study the emotional needs of depression, we also used the LDA which described before subdivided emotional needs into neutral, positive and three levels of negative emotions: negative I, negative II, and negative III. Negative I represent the health information with low mode such as feel lose, unhappy and so on and Negative III on behalf the most negative emotion with destructive behaviors such as suicide. The level of Negative II is between Negative I and Negative III. The specific explanation of the needs description type and needs type are shown in Table 2 and Table 3.

**Table 2:** Health information needs description classification

Category	Interpretation
C1 Etiology and Pathology	Describe or ask about the cause, rationale, and basics of depression, etc.
C2 Symptom	Describe or ask someone else to describe the symptoms of depression.
C3 Diagnosis	Describe the known diagnosis, or describe the symptom to ask if you have depression.
C4 Therapy	Describe the effects, procedures, or precautions of a treatments for depression such as psychotherapy and medication.
C5 Management	Describe or ask about the process of self-control and self-management of depressed patients.
C6 Complication	Describe or ask about depression-related mental illnesses such as two-way affective disorder, eating disorders, obsessive-compulsive disorders, post-traumatic stress disorder, drug abuse, or more.
C7 Prevention	Describe or ask how to avoid and prevent depression.
C8 Social activities	Describe or ask about social relationships, social disorders, social assessments, health insurance, and other social activities for people with depression.
C9 Education and Research	Describe and ask questions about depression education and research.

**Table 3:** Health information needs description classification

Category	Sub-category	Interpretation
Physiological needs		Maintaining of living material needs, such as food, drugs, etc.; and information needs such as disease-related websites, reports, and so on.
Affective needs	Positive	Positive psychological identity and emotional needs.
	Neutral	Neutral psychological identity and emotional needs.
	Negative	Negative psychological identity and emotional needs
Cognitive needs		Learning of skills or knowledge of depression。

There are three steps in the process of categorizing the question. First, using the LDA model and related paper get the needs type and the topic of each questions. Second, in order to ensure the accuracy of classification, we also carried out manual labeling. One coder performed a preliminary coding of all questions. Based on the results, inconsistent coding results between coder and the LDA model are discussed with the second coder and different coding questions are solved to get an agreement. Following the data coding, we computed the consistency between coder and LDA using Cohen’s kappa coefficients and the coefficients of healthy information needs description categories and healthy information needs type were 0.84 and 0.93. The results show that the data encoding is reliable [32].

## Results

### Statistics of data

Derived from the data we got from the Zhihu, the mean, median, mode and standard deviation of the essential features of the data were computed; see Table 4. Which contains the number of topics, answers, followers, the length of question, description and the question time.

**Table 4:** Basic characteristics of depression question

	Total number	Mean value	Median	Mode	Standard deviation
Number of Topics	5604	2.63	3	1	1.48
Length of question	42,264	19.85	17	14	10.05
Length of description	211,221	99.21	35	0	188.01
Number of Answers	5013	2.35	1	0	12.76
Number of Followers	19,283	9.06	2	1	124.62
Number of Viewers	3,792,120	1781.17	37	3	29,248.90
Question time	4522	2.12	2	1	1.04

**Fig. 2:**
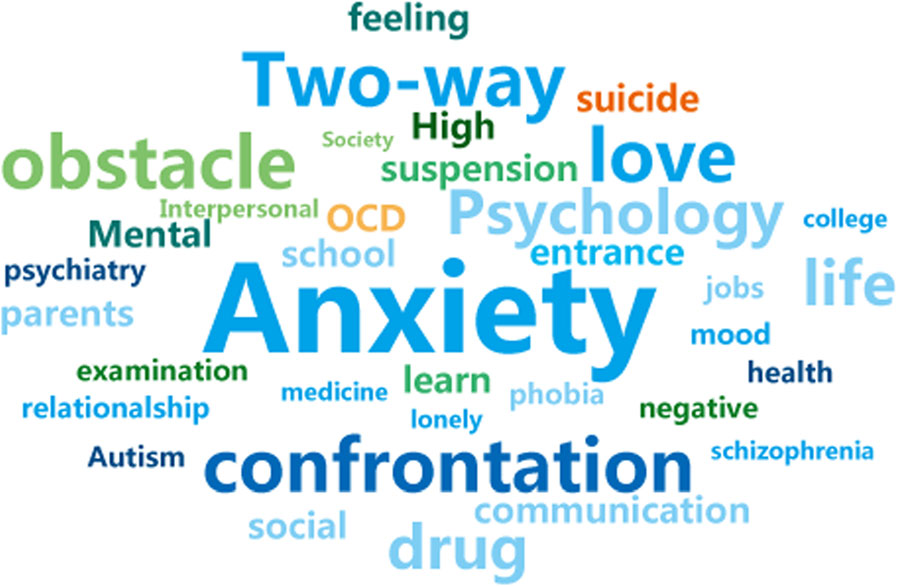
Top 50 hot topics in depression

At least one topic tag should be added to each question. The average number of tags for the topic of depression is 2.63 (SD = 1.48). The problems collected were located in topic of depressive so the tag “Depressive disorder” (*N* = 2743) and “Depression” (*N* = 742) are most. Depression is a mental disorder, so psychosocial topics are also popular, “mental illness” (*N* = 541) “psychology” (*N* = 523) and “psychology” (*N* = 427). After removing these five topics, the top 50 hot topics are shown in Fig. 2.

As noted above, the average length of the depression question is 19.85 (SD = 10.05), and the average length of the description of the requirement is 99.21(SD = 188.01). There are 1.8% questions whose length of description is equal or greater than 50. The length of the problem and the length of the description are weakly negatively correlated (corr = − 0.0273, *P* < 0.01). The longer the length of the question, the shorter the description will usually be. The average number of views on 2831 depression questions was as high as 1781.17 (SD = 29,248.90), but the number of followers and the number of answers were small, 9.06 (SD = 124.62) and 2.35 (SD = 12.76). Almost half of person with depression information needs choose to ask questions at night. The proportion of questioners in the morning, afternoon and evening is: 30.39, 20.45 and 49.16%.

### Health information needs description characteristics

**Fig. 3:**
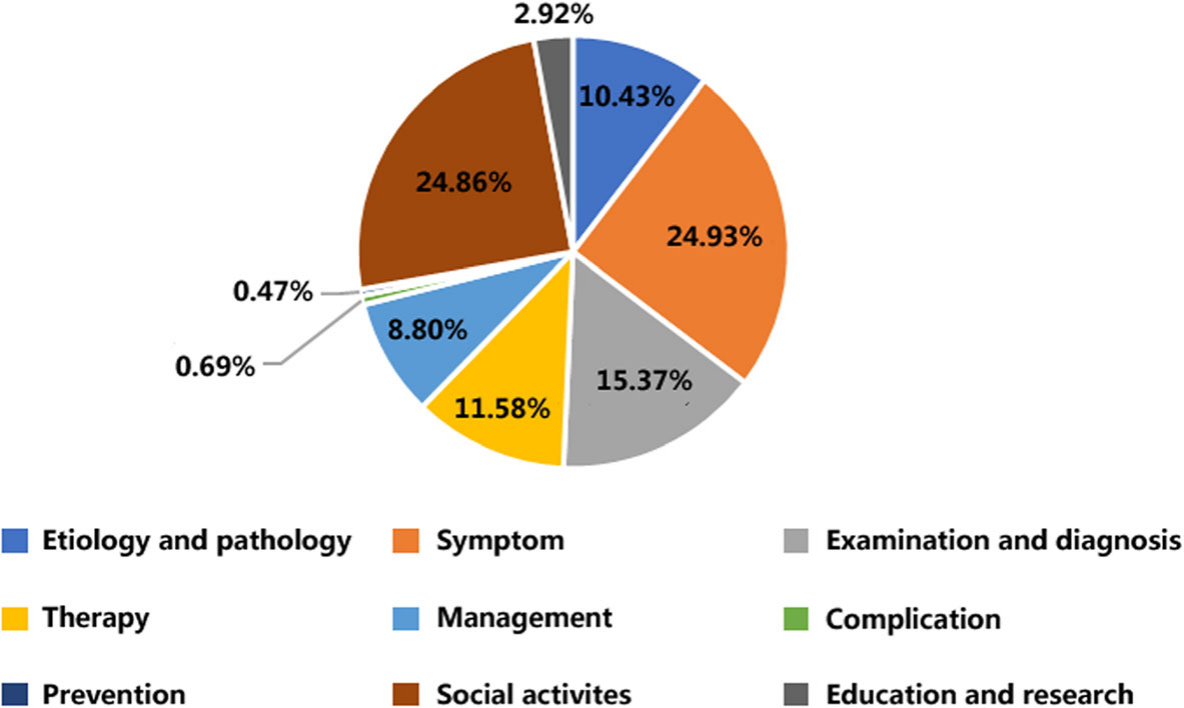
Depression needs description type

After coding the data and subject analysis, it was found that the health information needs were mainly focused on describing or asking about the symptoms of depression (24.93%) and social life (24.86%), see Fig. 3. Symptoms question are focused on asking if certain symptoms are suffering from depression such as low sleeping quality or suicidal tendency. Social life mainly includes boyfriends or girlfriends (Boyfriend is in severe depression, how can I help him?), relatives (Mother has major depression, how can I deal with her relationship?) and classmates/friends (friends have depression, often loses temper, what should I do?). Depression is a type of mental illness that requires more social attention than non-psychiatric diseases and so they pay more attention on the social life. The problems involving complications (0.69%) and prevention (0.47%) were all below 1%. The most frequently mentioned complications are anxiety, two-way affective disorder and obsessive-compulsive disorder.

The number of answers, followers, and views represent how much attention is paid to a question. In order to study whether there is correlation between nine different types of needs description and the user’s attention, we did a correlation analysis between different types of needs description and degree of attention in order to study their relations. The degree of attention is expressed by the sum of the number of answers, followers and views.

First, a descriptive statistical analysis of the attention of different needs descriptions is shown in Table 5. The question (topic) with highest level of attention was still symptomatic (average = 3493.60), and the standard deviation of the symptom class was also the largest (SD = 48,489.67), indicating that the participation of health information consumers participating in the symptom category was quite different. Prevention is the only type of requirement description with less than 100 attention (Mean = 32.46). Among the nine types of requirement descriptions, only etiology and pathology and Symptom showed a very weak positive correlation, while others showed very weak negative correlation. There is no significant correlation between the type of requirement description and the level of attention.

**Table 5:** The attention of different needs descriptions types

	Total number	Mean value	Standard deviation	Correlation coefficients
Etiology and Pathology	777,620	2690.73	27,042.462	1.21E-02
Symptom	2,253,370	3493.6	48,489.67	3.19E-02
Examination & Diagnosis	211,168	495.7	3242.89	-2.21E-02
Therapy	164,275	556.86	4102.65	-3.73E-04
Management	429,955	1762.11	24,735.53	−5.23E-03
Complication	3292	173.26	320.55	−4.70E-03
Prevention	422	32.461	30.87	−1.06E-02
Social activities	851,248	1287.81	15,738.96	−1.69E-02
Education & Research	18,098	223.43	753.13	−1.15E-02

### Health information needs type characteristics

**Fig. 4:**
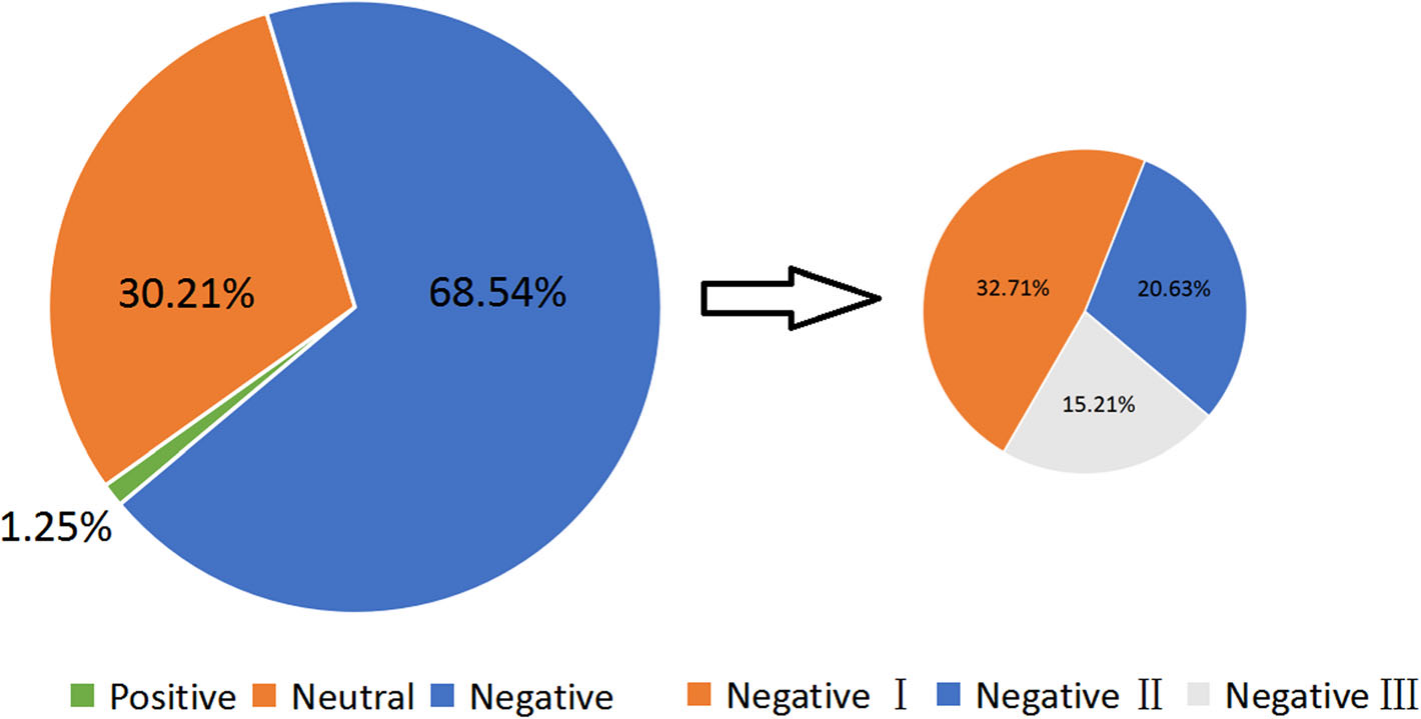
Depression affective needs type

Defining the type of health information needs helps to better understand the behavior of health information, and the type of demand for depression information is shown in the Fig. 4. Among them, the cognitive needs are the most dominant, accounting for 60.32%. The physical level has the least demand, accounting for only 2.24%. Most of them are asking about drugs and dietary information, such as: “What are the drugs that are effective for depression”, “What food is good when you take medicine in depression?”

**Fig. 5:**
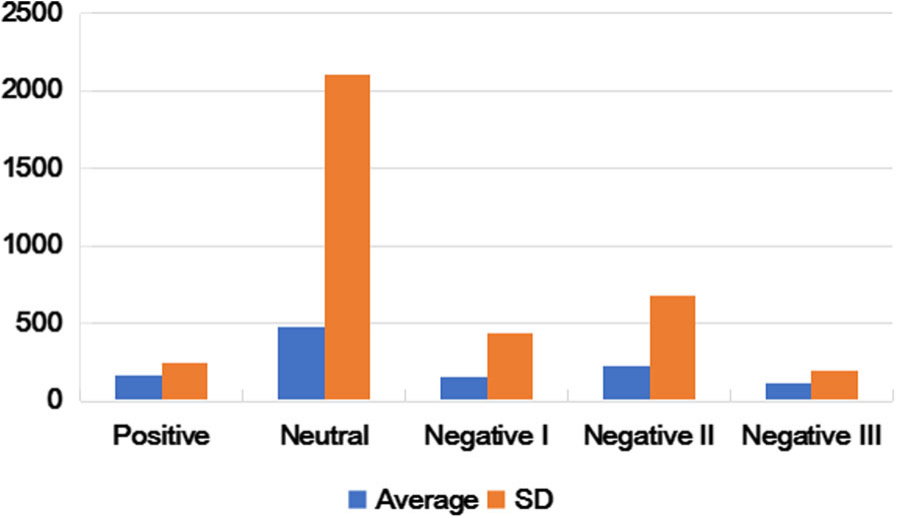
The attention of different affective needs type

Similarly, in order to study the correlation between the three types of health information needs and the degree of attention, we did a correlation analysis, as shown in the Fig. 5. Similarly, cognitive needs are the most concerned, with an average number of concerns of 4033.834 (SD = 33,138.66), as shown in the Table 6. The correlation between the three needs and the concerns of the questions is very low, and the cognitive need is the only one of the three needs that are positively related to the degree of attention (corr = 3.07E-04).

**Table 6:** The attention of different needs types

	Physiological needs	Affective needs	Cognitive needs
Total	4933	123,702	3,693,225
Mean	79.56	257.18	4033.83
SD	225.25	1232.43	33,138.66
Correlation coefficients	−1.25E-03	−1.13E-03	3.07E-04

In order to pay more attention to the emotional needs of patients with depression, we have subdivided 482 emotional needs into positive, neutral and negative I, negative II, and negative III. Among 482 questions, only 6 are positive emotions. For example: Are there any patients who have overcome depression together and move forward together? Negative emotions account for the largest proportion (68.54%), in which mild negative emotions accounts for the most. (for example: no energy now, and no interest in most of things, what should I do?). This is related to the persistent, hopeless, pessimistic and unsatisfactory characteristics of depressed patients. In order to explore whether higher concern is correlated with more negative emotions, the higher the concern in the community? We labeled positive, neutral, negative I, negative II, and negative III as − 1, 0, 1, 2, 3 and then did correlation analysis with attention, see Fig. 5. Neutral average attention is the highest (Mean = 475.90), negative III has the lowest attention (Mean = 112.95), which proves that intense emotions cannot lead to wider attention on the socialized question and answer platform.

## Discussion

### Health information consumers prefer to ask questions at night

There were 49.16% health information consumers chose to ask question at night. This is in line with the fact that people are more leisurely and depressed in the evening. In order to study the relationship between different post time and emotion, we labeled positive, neutral, negative I, negative II, and negative III as − 1, 0, 1, 2, 3 and then did correlation analysis with the time of ask question. There was weak correlation (corr = − 0.02882877) indicate there are more health information consumer tendency to ask negative question at night.

### Health information consumers pay more attention to the cognitive needs

Our study identified the cognitive needs are the most dominant, accounting for 60.32%. There are two reasons for this result. First, the social Q&A community serve as a platform for knowledge sharing for depression persons with social barriers. Second, the cognitive needs not limited by identify and professional knowledge compare with the specialized physical needs and the emotion needs which limited by the identify such as lover relative. At the same time, there were low correlation and high SD between the needs type and the concerns of the question. These results reflect the health information consumers have different needs for online health information and People have low trust in online health information, which makes it difficult to build close contact between information consumers and the online community.

### Health information consumers have a high degree of attention to symptoms

Our study found that the health information needs were mainly focused on describing or asking about the symptoms of depression (24.93%) and social life (24.86%). Depression is a type of mental illness that requires more social attention than non-psychiatric diseases and so they pay more attention on the social life. In addition, the symptomatic need also has the most attention and SD indicating that the participation of health information consumers participating in the symptom category was quite different.

## Conclusions

This paper analyzed the consumer health information needs by taking the depression information in Zhihu as an example. Using the LDA and manual, we divided the information needs description into nine categories. Also, we divided the information needs type into physiological needs, affective needs and cognitive needs depend on the Wilson’s model and we did divide the affective needs into five categories.

From the health information needs analysis of social Q&A community, it can be seen the people with depression pay the most attention on the symptoms and social life, and among the nine categories, there is not strong correlation between the levels of attention and the health information needs. As for the health information needs type analysis, the cognitive needs attract most attention by the health information consumers and the more negative emotion can’t get the more attention.

Overall, the health information providers in China, there are some suggestions as follows for a better health information environment based on our results. Frist, they should define health topics of interest for users and provide personalized health information services based on the degree of difference in each topic. Second, they can add a module which health information consumer can change the tags they’re interested.in. The change of tags can reflect the consumer needs and its trend. Last, they can implement a more profession management by adding the professional health knowledge.

Future research could be carried out in the following aspects. First, we should collect all the depression-related issues on the all social media in China, not just under the topic of depression in ZhiHu, for example Baidu ZhiDao. Second, further explore and pay more attention to the types of health information that are highly relevant in order to better serve health information consumers. Last but not least, use a better machine learning method to analyze the large amount of data, and better serve the health information consumers in China.

## Data Availability

The datasets used and analyzed during the current study are available from the corresponding author upon reasonable requests.

## Abbreviations

USA: United States
LDA: Latent Dirichlet Allocation
